# Pregnancy and health in the age of the Internet: A content analysis of online “birth club” forums

**DOI:** 10.1371/journal.pone.0230947

**Published:** 2020-04-14

**Authors:** Anna Wexler, Anahita Davoudi, Davy Weissenbacher, Rebekah Choi, Karen O’Connor, Holly Cummings, Graciela Gonzalez-Hernandez

**Affiliations:** 1 Department of Medical Ethics and Health Policy, University of Pennsylvania, Philadelphia, PA, United States of America; 2 Department of Biostatistics, Epidemiology, and Informatics, University of Pennsylvania, Philadelphia, PA, United States of America; 3 Department of Obstetrics and Gynecology, University of Pennsylvania, Philadelphia, PA, United States of America; Jagiellonian University Medical College, POLAND

## Abstract

**Background:**

Although studies report that more than 90% of pregnant women utilize digital sources to supplement their maternal healthcare, little is known about the kinds of information that women seek from their peers during pregnancy. To date, most research has used self-report measures to elucidate how and why women to turn to digital sources during pregnancy. However, given that these measures may differ from actual utilization of online health information, it is important to analyze the online content pregnant women generate.

**Objective:**

To apply machine learning methods to analyze online pregnancy forums, to better understand how women seek information from a community of online peers during pregnancy.

**Methods:**

Data from seven WhatToExpect.com “birth club” forums (September 2018; January-June 2018) were scraped. Forum posts were collected for a one-year period, which included three trimesters and three months postpartum. Only initial posts from each thread were analyzed (n = 262,238). Automatic natural language processing (NLP) methods captured 50 discussed topics, which were annotated by two independent coders and grouped categorically.

**Results:**

The largest topic categories were maternal health (45%), baby-related topics (29%), and people/relationships (10%). While pain was a popular topic all throughout pregnancy, individual topics that were dominant by trimester included miscarriage (first trimester), labor (third trimester), and baby sleeping routine (postpartum period).

**Conclusion:**

More than just emotional or peer support, pregnant women turn to online forums to discuss their health. Dominant topics, such as labor and miscarriage, suggest unmet informational needs in these domains. With misinformation becoming a growing public health concern, more attention must be directed toward peer-exchange outlets.

## Introduction

Pregnant women are increasingly turning to digital information to supplement their maternal health care. One study reported that 97% of women utilize online sources during pregnancy [1]; other research has reported similarly high (>90%) percentages [2–10]. More than just providing emotional support, digital sources influence women’s decision-making during pregnancy [1,4,5,11–15]. For example, some women use online information to validate or “get a second opinion” on their physician’s advice [1,11,16,17]. Given that online information has a real-world effect on behavior [18] and that women may be seeking Internet health information in lieu of obtaining it from their obstetrician—it is important to understand the kinds of information that women seek during pregnancy.

Prior work in the domain of digital health and pregnancy has focused on elucidating how and why women turn to digital sources during pregnancy [1,3,9,12,13,16,19–23]. However, these studies have a number of limitations. First, most have utilized self-report measures; yet the ways in which women *report* turning to digital health sources may be different than the way they actually *utilize* online health information in practice. Second, most studies have examined information-seeking during pregnancy as a whole, without looking at specific needs that may arise within each trimester. Third, there has been a dearth of research on information-seeking during the postpartum period, which is especially salient given that the American College of Obstetricians and Gynecologists (ACOG) recently identified “the lack of attention to [postpartum] maternal health needs” as an issue of particular concern [24].

An alternate approach to studying how women utilize the Internet during pregnancy is to examine the online content that women generate. Our prior work focused on gathering pregnancy-related information from a generic social media platform such as Twitter, whereby a cohort of pregnant women was identified via their self-reports of pregnancy and their timelines (all publicly available tweets) during pregnancy were analyzed in a case-control study of birth defects [25–27]. Other researchers have identified pregnant women based on their search queries, and determined their time-dependent search queries throughout pregnancy [28].

However, online pregnancy forums—which contain millions of posts generated by pregnant women—have largely gone unexamined. These forums, which are hosted on websites such as WhattoExpect.com and BabyCenter.com, have rapidly increased in popularity in the last decade. The sheer amount of data on these pregnancy forums offers a unique, untapped opportunity to examine how women seek online information from a community of peers during pregnancy. To our knowledge, the only prior general empirical study on these forums was conducted by Gui et al. (2017), who examined 200 online posts in each of three trimester-specific BabyCenter.com forums, utilizing a grounded theory research design [29]. Gui et al. (2017) found that women sought advice, informal and formal knowledge, reassurance, and emotional support; they also identified specific topics of support-seeking across the three trimesters.

At present, the most active discussion boards on pregnancy forums are due-date specific, (also known as “birth clubs”), where expectant mothers can connect with a community of peers undergoing a similar chronological pregnancy journey.[27] Based on publicly available data from WhatToExpect.com and BabyCenter.com, we estimate that there are currently over one million total posts (initial posts + replies) for each birth club forum (i.e., for each due date month and year, such as April 2019) across these two websites. Though the grounded theory approach utilized by Gui et al. (2017) offered researchers deep immersion in the data, it would be infeasible to manually code the hundreds of thousands of posts that comprise each birth club forum. Automatic and semi-automatic methods of analysis are thus required to utilize the data at scale.

The present study aims to apply automatic methods of language analysis (specifically, Latent Dirichlet allocation (LDA) topic modeling) to a much larger collection of birth club forum postings than what was used by Gui et al. (2017), in order to better understand the kinds of online information that women seek from their peers during pregnancy. We sought to discover whether women discuss health-related topics, and if so, which topics appeared most frequently. In addition, we aimed to differentially characterize the topics that women discuss across the three trimesters and the postpartum period to better understand how women’s information needs vary across these time periods.

## Methods

### Birth club forums

Currently, WhattoExpect.com (owned by j2 Global) and BabyCenter.com (owned by Johnson & Johnson) host the most active online forums for English-speaking women, as determined by number of members and posts. On both WhattoExpect.com and BabyCenter.com, posts are publicly viewable: membership is not required to access the forums, though users that seek to contribute a posting must become members by registering and creating a username. Usernames are not connected to social media accounts; thus the forums provide a level of anonymity. To obtain an approximate measure of the popularity of birth club forums across WhattoExpect.com and BabyCenter.com, we compared the number of total threads appearing in both birth club forums over the past five years (see supplementary material). Though BabyCenter.com was initially more popular, by fall 2018, the number of threads on WhatToExpect.com exceeded those on BabyCenter. Therefore, for the present study we drew our sample from WhatToExpect.com birth club forums. As all data (i.e., posts) for the present study were publicly available, we were advised by the University of Pennsylvania Institutional Review Board (IRB) that the project did not require review.

### Birth club dataset

First, we collected data for one WhatToExpect.com birth club forum (September 2018), and then included six other forums (January through June 2018) in our corpus. For each birth club forum, we narrowed our collection to an approximate one-year period that included all three trimesters plus the postpartum period. For our analysis, we considered only initial posts and not data from the entire thread, as initial posts most clearly reflected the goal of the study: to understand the kinds of information that women seek on online forums. Replies to initial posts sometimes digressed and included divergent topics.

In order to distinguish topics throughout the pregnancy from those that arise primarily during a specific trimester, we divided all posts in our corpus into four time periods, corresponding to each of the three trimesters and the postpartum period. Given that we could not know each woman’s delivery date, for the purposes of the study we fixed the delivery date to the end of the birth club month, and counted trimesters and the postpartum period in blocks of three months (90 days) back from that date. Thus, for the September 2018 forum, birth was assumed to have occurred on September 30, and the ensuing 90 days (October 1 through December 29, 2018) was considered as the postpartum period. Working backward in 90-day blocks, posts were divided into the third trimester (July 3 through September 30, 2018), second trimester (April 4 through July 2, 2018), and first trimester (January 4 through April 3, 2018). We chose this normalized division (over using the middle of the month as a due date, for example) as it provided the clearest distinction between the main topics, particularly between the third trimester and the postpartum period. We assigned each post to a unique corresponding trimester based on its date of publication.

This study was deemed exempt from review by the University of Pennsylvania Institutional Review Board due to the fact that it did not meet the regulatory definition of research with “human subjects,” as the data was obtained from publicly available sources. In addition, we verified that the study methods were in compliance with the WhatToExpect.com Terms of Use (https://www.whattoexpect.com/terms-of-use/).

### Topic modeling and labeling

We utilized automatic natural language processing (NLP) methods to capture the discussed topics. Specifically, we used a form of topic modeling known as Latent Dirichlet Allocation (LDA), which assumes that each document in a large dataset is comprised of sub-topics that are represented by the words they contain. By analyzing the frequency of word appearance in relation to other words, the LDA algorithm can discover a “bag of words” that have a high probability of appearing together. If the model has performed optimally, the bag of words can be identified (or “labelled”) by a human coder as relating to a specific topic. Importantly, the topics are not pre-defined, but rather are discovered during the algorithm’s training stage.

The performance of topic modeling algorithms is strongly determined by the choices made to pre-process the documents and to optimize the hyper-parameters of the training algorithms. Because some words occur in most documents and do not help to identify distinct topics, we removed the following in the pre-processing stage: (a) all digits; (b) all hyperlinks to external websites; (c) common words such as articles and prepositions (e.g. a, the, of, always, etc.) and (d) generic words related to pregnancy, such as baby, born, days (see supplementary material for complete list of excluded “stop words”).

Notably, the number of topics in an LDA model is always finite, and the total number of topics desired is set as an initial parameter. To determine the optimal number of topics for our model, we trained multiple models—with 20, 35, 50, 80 and 150 topics—over all initial posts in the March 2018 and September 2018 forums and compared the resulting sets of words manually. Annotators inspected the word clusters in each set of topics, and chose the 50-topic model given that the word clusters were distinct enough to be assigned a "label" for each topic. With a number of topics lower than 50, the topics failed to capture the full diversity of subjects discussed in the forums. With a number higher than 50, the main topics were not adequately represented as unified topics but rather split into multiple related sub-topics.

The topic modeling system runs through the documents many times during the training phase; topics become more distinct with each additional iteration. We found that 200 iterations were adequate for our model, as additional iterations did not result in noticeable differences in the topics learned. Thus, with these hyper-parameters selected—50 topics and 200 iterations—we computed topics on all initial posts contained in our corpus.

To define topic labels, two annotators independently inspected the 15 words that were the most representative of the resulting 50 topics. Any disagreement in topic labels was resolved through discussion and by reviewing the ten posts most strongly associated with a given topic. A third annotator confirmed the final topic labels through examination of the top 100 posts associated with each topic, occasionally making minor refinements to the wording of the topic label. Next, all three annotators worked together to group the topics into overall categories and subcategories.

## Results

**Fig 1:**
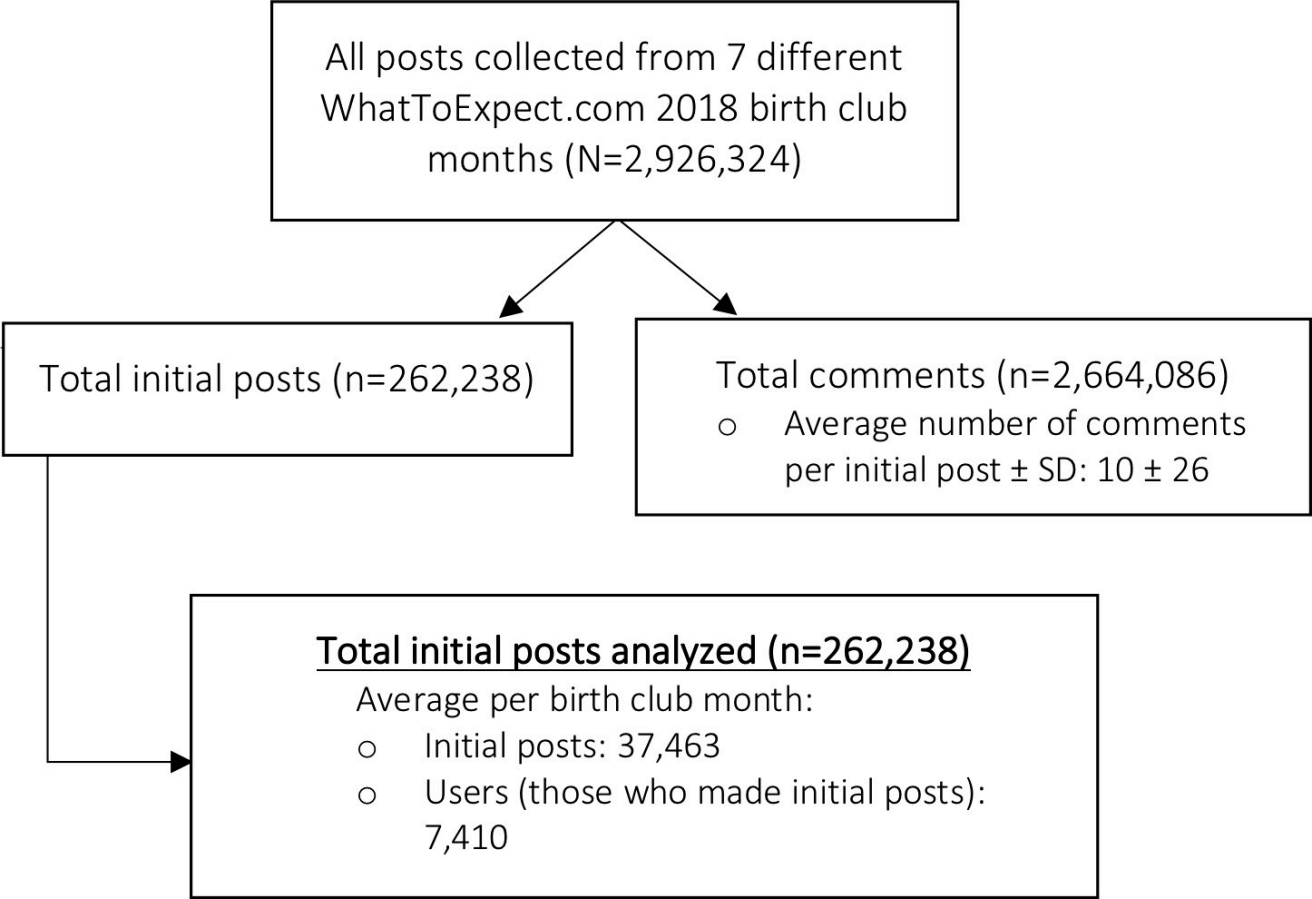
Data collection flowchart.

**Fig 2:**
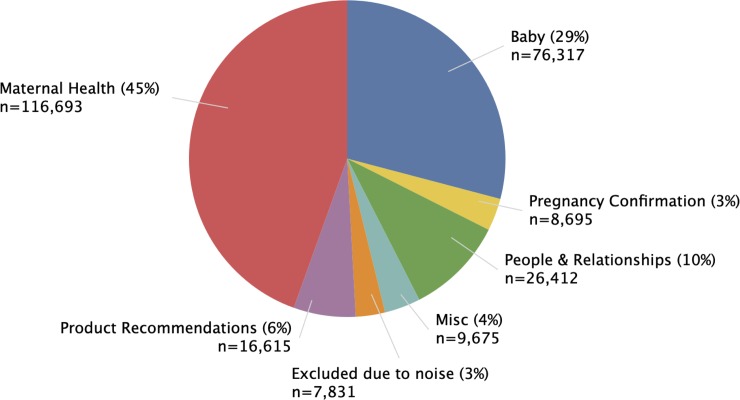
Distribution of posts by overall topic category.

We collected 2,926,324 posts across the seven WhatToExpect.com birth club forums (**Fig 1**). Of those, 262,238 represented initial posts and the remainder were follow-up posts (i.e., all posts following the initial post on a given thread). Initial posts were analyzed (**Fig 1**), for which our model generated 50 distinct word-clusters. Six word-clusters were excluded from our analysis as they were noisy and did not constitute semantically coherent sets. However, annotators successfully labeled 44 word-clusters, and grouped the resulting topics into subcategories and overarching categories (**Fig 2**).

**Fig 3:**
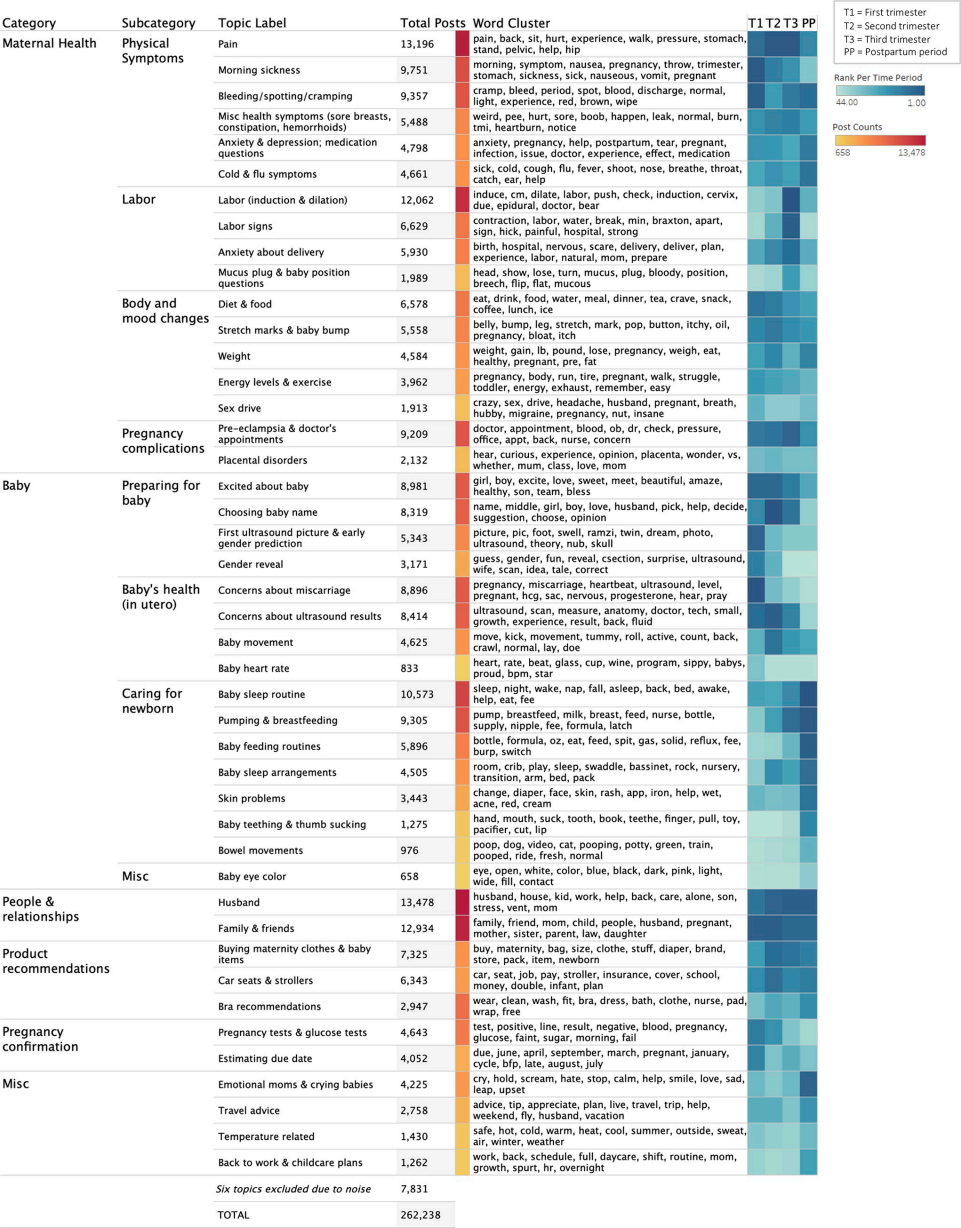
Topics of discussion on online pregnancy forums. From left to right: Overall topic categories, subcategories, and topic labels. Total posts per topic are visually represented by yellow-red frequency heat map. The word cluster column contains the top 12 words generated by our algorithm, per topic. The teal-blue heat map depicts topics in rank order within each time period.

**Fig 3** presents a detailed view of the 44 most dominant topics across all trimesters and their related word clusters. All topics were analyzed by their frequency of appearance using several methods. First, we examined the overall topic frequency across all time periods as a whole (middle bar, **Fig 3**). Next, we analyzed the most frequent topics that appear within each of our four time periods (three trimesters and postpartum). In doing so, we sorted topics from highest-to-lowest in rank order, so that the most frequently-appearing topic in a time period was given a rank of one, the second a rank of two, etc. We analyzed all topics by their rank order across all trimesters (right column, **Fig 3**).

As shown in **Fig 2**, the largest category of topics was related to maternal health (45%), followed closely by topics related to the baby (29%), and people/relationships (10%). Within the category relating to maternal health, physical symptoms (including nausea, pain, and bleeding) was the most dominant subcategory (**Fig 3**), followed by a subcategory relating to labor (including induction/dilation, labor signs, and anxiety about delivery). Body and mood changes, pregnancy complications, and concerns about miscarriage comprised the remainder of subcategories. When examining the overall count of topics across all time periods, both pain and labor topped the list, as indicated by the dark red squares (middle column, **Fig 3**). An analysis of the topics by rank order across the trimesters (right column, **Fig 3**), shows the dominance of certain topics by trimester: concerns about miscarriage, for example, appear frequently in the first trimester (as indicated by the dark blue square) but are hardly discussed in the ensuing time periods (as reflected by the lighter blue squares). Similarly, topics relating to labor appear most frequently in third trimester, whereas discussions of pain are prevalent across all four time periods.

Within the category relating to babies, caring for the newborn was the most frequent subcategory, which included specific topics relating to baby sleeping and feeding routines, as well as breastfeeding. Overall, “baby sleep routine” was one of the most dominant topics across all time periods (middle bar, **Fig 3**), and topics related to caring for the newborn are ranked highly in the postpartum period (right column, **Fig 3**). Other subcategories relating to baby were preparing for baby (including excitement about baby, ultrasound pictures, and gender reveal) and baby’s health in utero (including concerns about ultrasound results and baby movement); these appear most prominently in the first and second trimesters (right column, **Fig 3**).

The category relating to people/relationships only contains two topics: husband and family/friends. However, this category is notable in that these two topics appear frequently overall (middle column, **Fig 3**) as well as frequently within each individual time period (right column, **Fig 3**). Example posts in these topics include questions about when to share a pregnancy announcement, discussions of how a new baby will impact family dynamics, sharing of household labor, and requests for advice on relationship issues.

The category relating to pregnancy confirmation indicates that some women turn to specific birth club forums very early on, to discuss pregnancy test results, to share struggles regarding conception, or to estimate their due date. The category relating to product recommendations shows that most women are looking for advice on clothing (maternity-wear, baby clothes, bras) and baby transport options; the topics here are most dominant in the second trimester, when women are most likely to begin needing maternity clothes and when they start preparing for baby. The final category consists of several miscellaneous topics, regarding emotions, travel advice, temperature, and going back to work. Although the latter topic has only a small number of posts, a significant number of posts in the “husband” category are related to going back to work, as evidenced by the word cluster in that topic and our manual examinations of representative posts; it is therefore likely that our model captured many work-related posts in that category.

**Fig 4:**
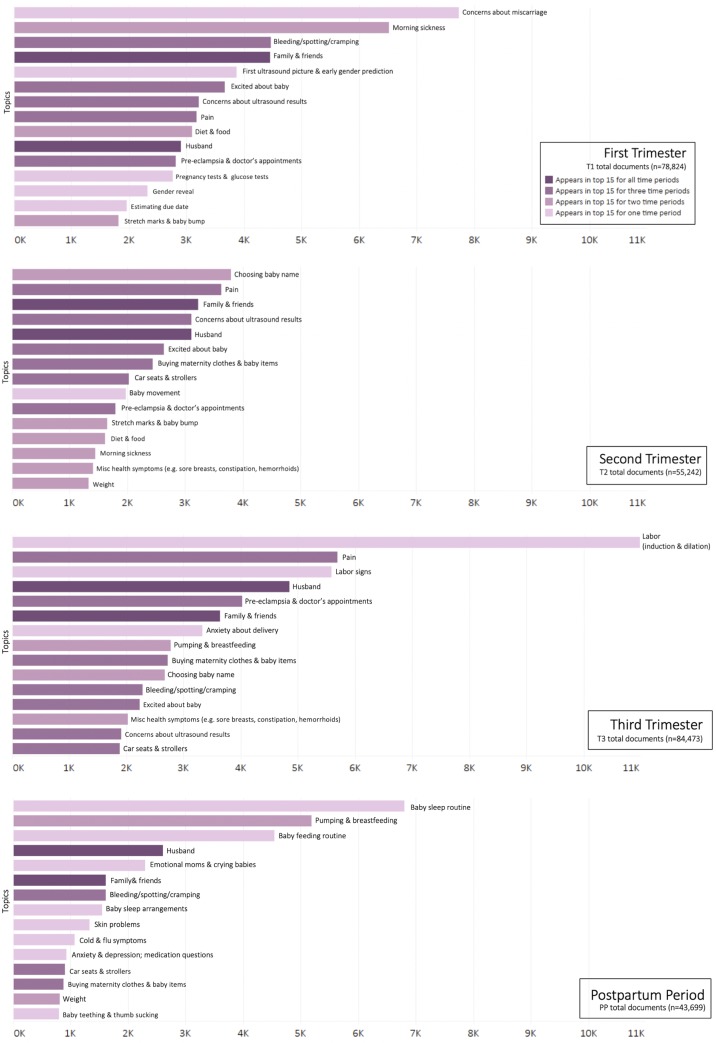
Top fifteen topics for each time period. Topics that are prevalent in one or more time periods are notated by darker colors(s). Number of posts per time period is reported (n). Bars represent observed value.

Finally, as rank order obscures the numerical differences between the most frequent topics, we also depict the numerical counts of the top 15 ranked posts per time period (**Fig 4**). All time periods except for the second trimester have a topic that clearly dominates. In the first trimester, topics related to concerns about miscarriage, morning sickness, and bleeding/cramping (which is likely also related to miscarriage) appear most frequently. In the third trimester, labor-related topics and pain are the ones that most frequently appear. Notably, not only is “labor (induction/dilation)” the single most frequent topic when looked at in any individual time period, but when considered along with closely related topics in this trimester (“labor signs” and “anxiety about delivery”), discussions of labor total approximately 20,000 posts—an amount that dwarfs all other posts in individual time periods. Finally, two topics—husband and family/friends—appear in the top 15 across all time periods (darker bars, **Fig 4**).

## Discussion

The results of this study demonstrate that more than just emotional or peer support, or even product recommendations, pregnant women are turning to online forums to discuss their health. They post about topics such as bleeding, cramping, pain, labor, and morning sickness. They also turn to the forum to discuss worries about their baby’s health, such as fears of miscarriage, baby’s heart rate, and abnormal ultrasound results. One of the most striking findings from this study is that two topics, miscarriage and labor, are by far the most dominant topics within specific time periods (first and third trimester, respectively).

Our findings are largely distinct from previous work that has directly queried women (via interviews or surveys) about topics of information-seeking during pregnancy, which has found that the most common information sought online during pregnancy is related to fetal development, nutrition in pregnancy, stages of childbirth, and pregnancy complications [1,6,7,9,12,30–35]. Aside from the topic relating to childbirth, which is analogous to our “labor” topic, the remaining topics either did not arise in our findings (i.e, fetal development) or were not dominant in specific trimesters. The topics found in our study, however, are largely similar to those from Gui et al. (2017), who analyzed trimester-specific forums on BabyCenter.com, and overlap to some extent with a study examining pregnant women’s search queries [28].

Thus, it seems that the few studies examining the actual content of either forum conversations or search queries have largely converged on similar findings, whereas studies that have asked women about their online information-seeking behavior have resulted in a second set of findings. There are several possible explanations for this divergence. First, there may be genuine differences between the kinds of information that women glean from apps or static websites as compared to what they post on online forums. For example, if an information need, such as stages of fetal development, is satisfied by an app, women may be less inclined to post questions about it on an online forum. Second, the interview and survey studies varied in query methodology. Women may have responded differently if a question encompassed use of digital sources as a whole, as compared to a question about Internet searches. Third, there may have been a recall bias in interview and survey studies, resulting in participants not accurately recalling their most commonly searched topics. Fourth, there could be a selection bias in that women who post on online forums are interested in different topics than a random sample of pregnant women.

Notably, pregnant women are likely encountering forum discussions even if they have not joined an online community. As noted above, the vast majority of women are turning to online information during pregnancy, and the way they do so is primarily through the use of search engines [1,3,5,6,11,19,36]. While generic search terms such as “pregnancy” or “miscarriage” may yield results from static websites, natural language queries such as “is shooting pain during the second trimester normal” may generate links to forum posts. Indeed, one study that analyzed search engine results for pregnancy-related queries found that natural language search terms (i.e., “my baby is moving less”) mostly resulted in forum posts, whereas general terms such as “fetal movement” mostly generated results for static webpages and articles [37]. In other words, women who use natural language terms in their search queries will likely stumble across forum posts at some point—or possibly even quite frequently—during their pregnancy.

This is especially concerning, given that as many as 70–75% of pregnant women do not speak to their health care providers about information retrieved from the Internet [23,30,34]. Furthermore, one recent study analyzing the quality of online information in Internet discussion forums in pregnancy found that 24.3% of responses lacked credible evidence and 5.5% were potentially harmful [38]. Yet women typically perceive online pregnancy information to be reliable and of high quality [1,3,5,9,30,34], and use it to inform their health care decision-making [4,5,12,14]. Thus, while information retrieved from online forums may be influencing women’s health choices, this behavior may not always be transparent to the health care provider.

To the extent that the most dominant topics in **Fig 3** can be interpreted as unmet informational needs, our study has a number of practical implications. First, pregnancy literature typically provides women with information about common symptoms and body changes, such as weight gain, nausea, stretch marks, abdominal and back pain, and energy levels. There is little information, other than “call your doctor,” regarding pregnancy complications, such as miscarriage, pre-eclampsia, or abnormal ultrasound results [17]. On the one hand, given that the majority of women will not experience these complications, this strategy may avoid causing undue concern. On the other hand, in the age of the Internet, the lack of readily available information about “atypical” pregnancies ensures that those experiencing complications will likely go online to search for information. Women may do this either in lieu of contacting their health providers, to prepare for appointments, or even to “double-check” information provided to them by their physician [1,11,16]. Health care providers should recognize that they may no longer be the primary source of health information during pregnancy, and professional societies may want to consider providing reliable information online regarding pregnancy complications.

Second, the total number of posts related to labor points to a critical information gap in this area. Currently, aside from optional childbirth classes, there is little to prepare women for labor, both from an emotional or informational perspective. Labor is perhaps the area that generates the most uncertainty and anxiety during pregnancy, especially for first-time mothers: specific posts in our sample included questions about how to discern when one is in labor and/or when one’s water has broken; when to go the hospital; how to induce labor; and how to cope when a birth is proceeding differently than expected. Given the significant informational and support needs related to labor, it is not surprising that studies have consistently found benefits of companionship during the labor process (e.g., such as that provided by a doula) [39–41]. The results of our study, which indicate that there is still a significant unmet informational and emotional support need, support ACOG’s recommendation of incorporating support personnel such as doulas during labor, as well as other suggestions to have labor support routinely provided by healthcare systems [41–43].

Third, the number of topics in the postpartum period related to newborn care—particularly with regard to feeding and sleeping routines—indicates another crucial information gap. Here, too, there is often little information about newborn care provided to women in the lead-up to birth; after delivery, women must shift their questions to a new care provider, the pediatrician. Indeed, in the immediate postpartum period—a time when women are experiencing significant physical and psychological challenges—the provider who has supported them throughout pregnancy is no longer responsible for care, and will not typically see them for six weeks. From the women’s perspective, pregnancy, labor and newborn care are a continuous process; this continuity, however, is not mirrored by health care systems [44]. The results from our study suggest that additional information and emotional support should be provided to women during the postpartum period.

This study has a number of limitations. The topics in **Fig 2** do not indicate *all* the topics discussed during pregnancy but rather indicate word clusters that our model determined had the strongest correlations between words. Posts that were very general in nature, or those that did not include unique words, may not have been detected by our model. In addition, some word clusters may not have arisen due to our list of excluded words (e.g., the exclusion of “born” and “section” likely precluded word clusters related to birth announcements and caesarian sections). Furthermore, given that our model is based on associations between words, some of our topics contain multiple sub-topics with related words. For example, the topic related to preeclampsia and blood pressure also contained general posts related to doctor and doctor’s appointments, and the topic related to bowel movements was mostly related to baby but also contained posts regarding exposure to cat feces during pregnancy. Finally, as noted in the methods section, we could not capture the exact date of birth for each poster, thus we relied on estimates of trimester timeframes.

Because WhatToExpect.com displays limited information about each user, we could not determine users’ genders, locations, or ages. Nor could we determine the relative percentages of nulliparous and primiparous women. All posts we reviewed appeared to be from heterosexual women, which is in-line with the findings of Gui et. al (2017)’s analysis of the BabyCenter forum. It was also our impression that the majority of users are from the United States, though some users appear to be living in Canada, the United Kingdom, and Australia, as evidenced by the usage of certain words (e.g., “pram” and “nappy”) and mentions of different health care systems.

To our knowledge, this study is the first to provide a large-scale, big data analysis of online pregnancy forums. While several previous studies have manually examined forum posts relating to specific topics—such as vaginal birth after caesarean section [45], multiple sclerosis [46], and vaginal breech birth [47]—the present study examined 262,238 posts from seven different birth club months, yielding a general topical portrait of all posts. By conducting frequency analyses both overall and within four different timeframes, we were able to highlight dominant topics overall as well as those that were trimester-specific.

In some sense, the fact that individuals are utilizing online forums to seek out health information is nothing new [18]. In the realm of pregnancy, however, the data is clear: the vast majority of women are utilizing digital health information during pregnancy and are using that information to inform their decision-making, all while not necessarily mentioning this process (or retrieved information) to their healthcare provider. It is crucial that more scholarly attention be directed to peer-exchange outlets such as online pregnancy forums, especially as “misinformation” is becoming increasingly recognized as a matter of public health importance [48–50]. Professional societies may want to consider providing guidelines to health care providers regarding how to navigate their patients’ use of online information. This study, which outlines the topics of discussion on online pregnancy forums, should provide the first step in informing that pathway forward.

## Supporting information

S1 Data (PDF)
