# Supplementary material for: Pregnancy and health in the age of the Internet: A content analysis of online “birth club” forums
Source: PLoS One. 2020 Apr 14;15(4):e0230947. doi: 10.1371/journal.pone.0230947 (PMC7156049; doi:10.1371/journal.pone.0230947)
Supplement: S1 Data — (PDF) [file pone.0230947.s001.pdf]

Supplemental Figure 1. BabyCenter.com & WhattoExpect.com: Approximate number of threads for birth month group (2014-2019)

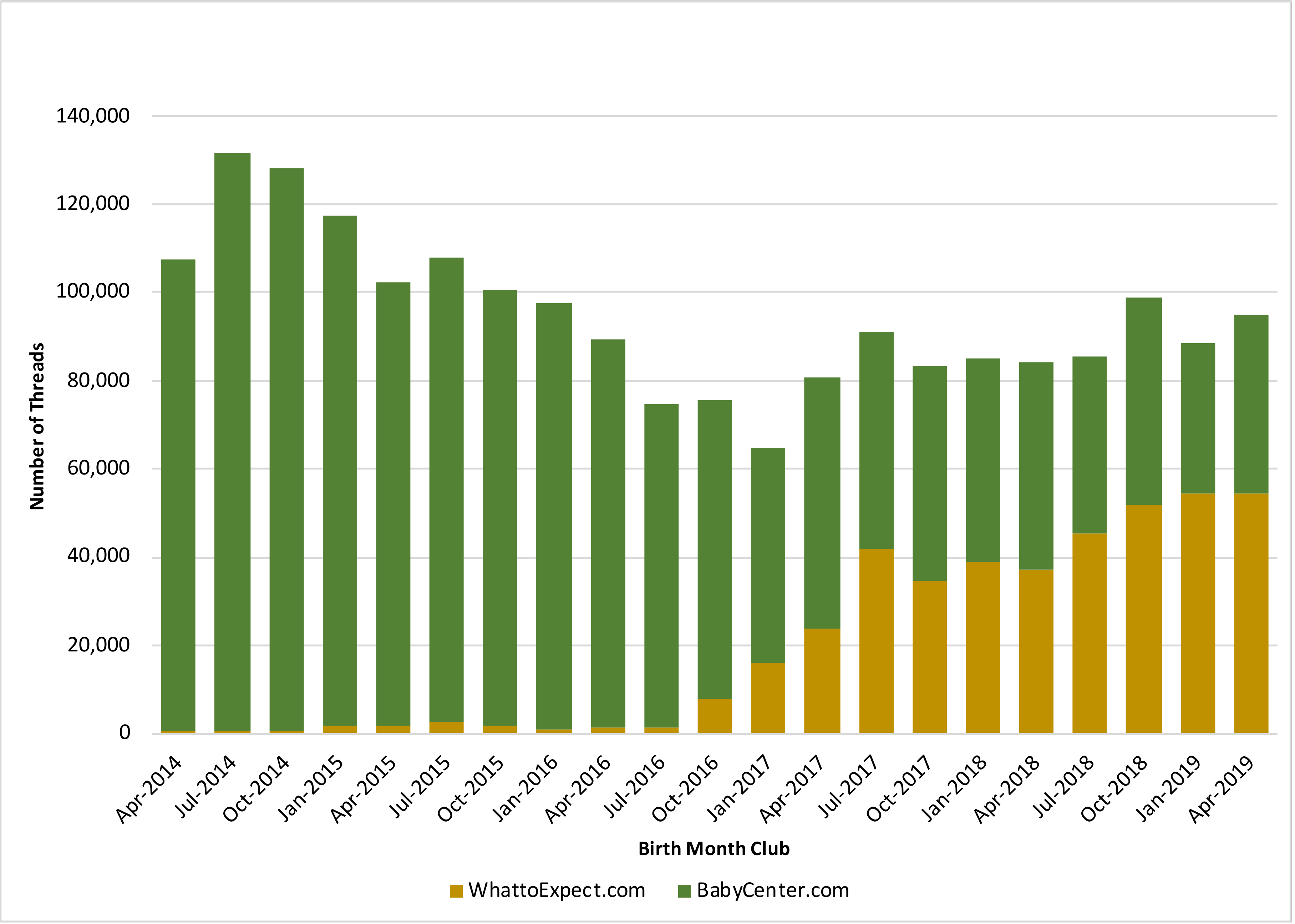

Supplemental Figure 1. To obtain a measure of the popularity of birth club forums across WhattoExpect.com and BabyCenter.com, we counted the number of total threads appearing in birth club forums over the past five years. Each initial post was counted as a single thread, regardless of the number of follow-up comments. Because these forums continue to be active years after childbirth, prior years tend to have a greater number of cumulative posts. Therefore, we restricted our count to the number of threads appearing in a one-year period, dating form the start of the first trimester to three months postpartum. Thus, for the April 2015 forum, we used publicly available data to count the number of posts appearing from August 1, 2014 through July 31, 2015. Data was obtained from the two websites quarterly (i.e., every fourth birth club month) for the last five years, from the April 2015 birth club through the April 2019 birth club.

Supplemental Figure 2. Left: sample screenshot from the WhatoExpect January 2018 birth club month forum. Right: Sample thread from the forum, depicting the initial post and follow-up responses. Usernames have been redacted.

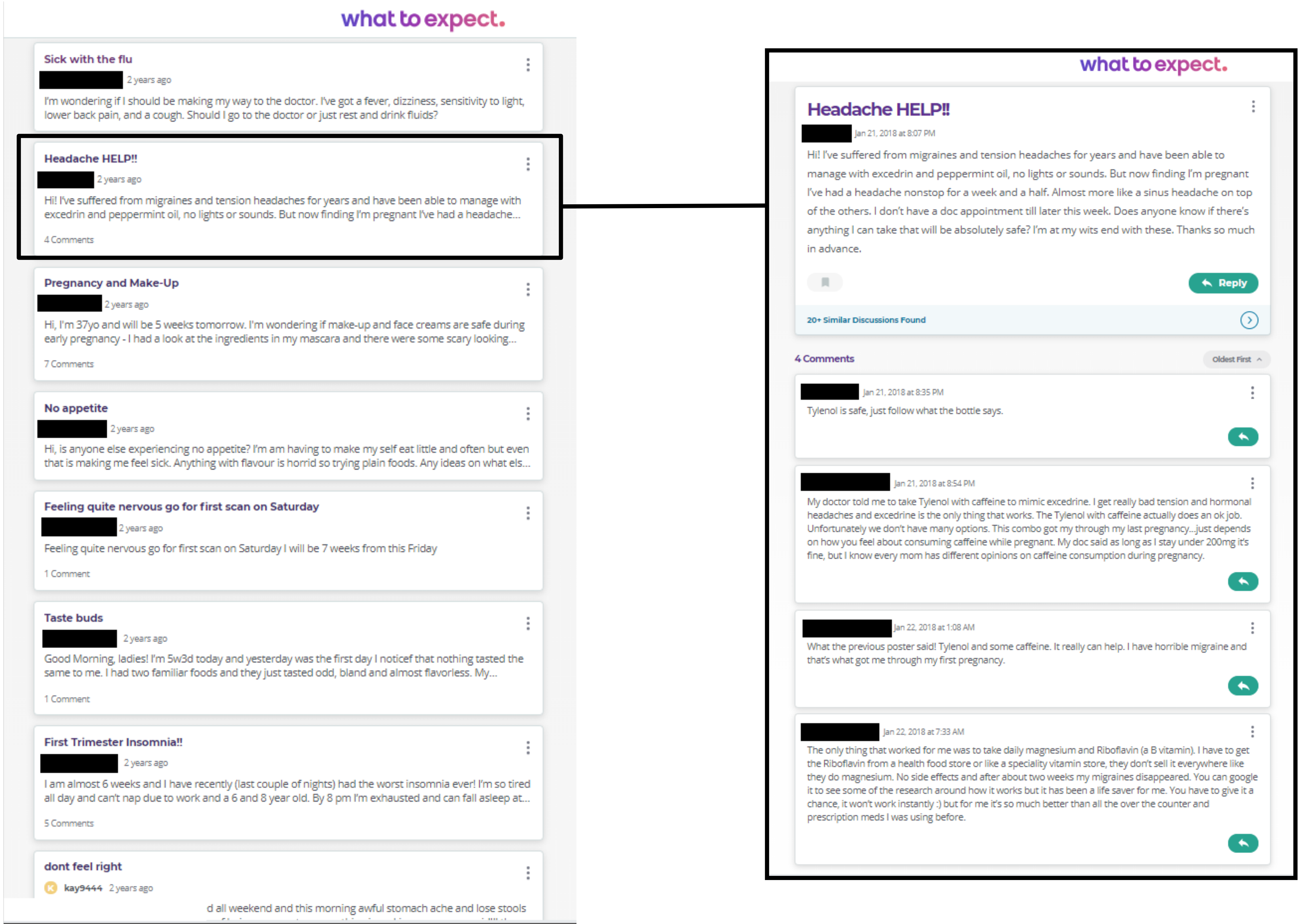

Supplemental Figure 3. It is a standard practice when using Latent Dirichlet allocation to remove "stopwords" or common, contentless words in a corpus as a preprocessing step [1]. It has been shown that beyond very high probability terms, the effects of stoplists on training are limited [2], but that a set of extremely frequent terms may overwhelm the model—whereby all topics look very similar, simply reflecting the frequent terms—and reduce how well the model fits contentful (meaningful) terms that better highlight the focus of a user post. Thus, as a preprocessing step, we opted to remove terms that occur very often in the collection of user posts, eliminating words such as “the” and "would" that are generally very frequent in English, as well as terms that are not contentless but rather are ubiquitous in the forum (such as "baby" or "pregnancy").

1. Blei DM, Ng AY, Jordan MI. Latent Dirichlet Allocation. J Mach Learn Res. 2003; 993–1022.
2. Schofield A, Magnusson M, Mimno D. Pulling Out the Stops: Rethinking Stopword Removal for Topic Models. 2017; 432–436. doi:10.18653/v1/e17-2069

List of stop words

'
"
-
!
!?
#
###
$
%
&
(
)
*
,
.
..
...
:
;
?
?!
??
@
[
]
^
`
``
{
}
+
<
>
a
able
about
above
absolutely
actually
after
again
against
ago
ahead
all
almost
alot

already
also
although
always
am
an
and
another
any
anymore
anyone
anything
anyway
are
arent
aren't
around
as
at
away
babies
baby
baby\xe
bad
barely
basically
bc
be
because
been
before
being
below
best
better
between
bit
born
both
bout
but
by
can
can\'t
can\xe
cannot
cant
can't
cause

come
completely
could
couldn\xe
couldnt
couldn't
currently
daily
date
dates
day
days
definetely
did
did\nt
did\n't
didn\'t
didn\xe
didnt
didn't
do
does
does\xe
doesn\t
doesn\'t
doesn\xe
doesnt
doesn't
doing
don\'t
don\xe
done
dont
don't
down
during
each
earlier
early
eighter
either
else
end
enough
entire
especially
etc
even
eventually

ever
every
everyone
everything
exactly
except
extremely
f\x
far
feel
few
fifth
finally
fine
first
firts
for
forward
found
fourth
from
further
get
give
go
gosh !!
ha
had
hadnt
hadn't
hah
haha
hahaha
has
hasnt
hasn't
have
haven\'t
haven\xe
havent
haven't
having
he
he\'s
he\xe
he'd
he'll
her
here

heres
here's
hers
herself
hes
he's
him
himself
his
honestly
hopefully
hour
hours
how
however
hows
how's
hrs
i
i\'d
i\'ll
i\'m
i\'ve
i'\ve
i\xe
i'd
if
i'll
im
i'm
in
into
is
isn\'t
isn\xe
isnt
isn't
it
it\'s
it\xe
its
it's
itself
i've
just
keep
kind
kinda
kinds

last
later
leA1164ast
least
less
let
lets
let's
like
likely
literally
little
ll
'll
lo
lol
loll
lool
lot
lots
low
-lrb-
-lsb-
'm
make
many
may
maybe
me
might
mine
minute
minutes
moment
month
months
more
moreover
most
mostly
much
must
mustnt
mustn't
my
myself
need
never
new

next
no
noone
nor
normally
not
nothing
now
obviously
odd
of
off
often
oh
ohh
ohhh
ok
okay
okayy
old
on
once
one
ones
only
or
other
others
ouch
ought
our
ours
ourselves
out
over
own
part
parts
per
personally
please
probably
put
rather
re
ready
recently
return
right

-rrb-
-rsb-
's
same
say
second
seconds
section
sections
see
seem
seems
seriously
several
shant
shan't
she
she\'s
she\xe
she'd
she'll
shes
she's
should
shouldnt
shouldn't
side
since
sixth
so
some
someone
something
somethings
sometime
sometimes
soon
sorry
start
stay
stil
still
such
super
sure
tell
th
than
thank

that
that\'s
that\xe
thats
that's
the
their
theirs
them
themselves
then
there
there\xe
theres
there's
these
they
they\xe
they'd
theyll
they'll
theyre
they're
theyve
they've
thing
things
third
this
those
though
three
through
throughout
till
time
times
to
today
together
tomorrow
ton
too
total
totally
tried
tries
try
trying

twice
two
under
unfortunately
unless
until
up
us
use
used
uses
using
usually
ve
very
wait
want
wanted
wanting
wants
was
wasn\'t
wasn\xe
wasnt
wasn't
way
we
we\xe
we'd
week
weeks
well
we'll
were
we're
werent
weren't
we've
what
whatever
whats
what's
when
whenever
whens
when's
where
wheres
where's

which
while
who
whole
whom
whos
who's
why
whys
why's
will
with
within
without
wks
won\'t
won\xe
wondering
wont
won't
word
would
wouldn\'t
wouldn\xe
wouldn\xe
wouldnt
wouldn't
xe
xef\xb
xf
yeah
year
years
yes
yesterday
yet
you
you\re
you\'re
you\xe
youd
you'd
youll
you'll
your
youre
you're
yours
yourself

yourselves
youve
you've
